# Preference for Service Delivery for Long-Acting Pre-exposure Prophylaxis for HIV Infection Among Pregnant and Breastfeeding Women in South Africa and Botswana

**DOI:** 10.1007/s10461-025-04751-6

**Published:** 2025-05-21

**Authors:** Lindsey de Vos, Aamirah Mussa, Elzette Rousseau, Michael Strauss, Gavin George, Prisca Vundhla, Avuyonke Gebengu, Maipelo Tsuaneng, Lefhela Tamuthiba, Aratwa Tumagole, Neo Moshashane, Chelsea Morroni, Remco P. H. Peters, Chibuzor M. Babalola, Jeffrey D. Klausner, Dvora Joseph Davey

**Affiliations:** 1 Research Unit, Foundation for Professional Development, East London, South Africa; 2 Botswana Harvard Health Partnership, Gaborone, Botswana; 3 Usher Institute, University of Edinburgh, Edinburgh, UK; 4 Desmond Tutu HIV Centre, University of Cape Town, Cape Town, South Africa; 5 Health Economics and HIV and AIDS Research Division (HEARD), University of KwaZulu-Natal, Durban, South Africa; 6 Centre for Reproductive Health, University of Edinburgh, Edinburgh, UK; 7 Department of Population and Public Health Science, University of Southern California, Los Angeles, CA USA; 8 Division of Infectious Diseases, Geffen School of Medicine, University of California Los Angeles, 911 Broxton Ave., # 301, Los Angeles, CA 90024 USA; 9 Division of Epidemiology and Biostatistics, School of Public Health and Family Medicine, University of Cape Town, Cape Town, South Africa

**Keywords:** Discrete choice experiment, Pregnant, Postpartum, PrEP, Women, Africa

## Abstract

HIV particularly affects women during pregnancy and postpartum, where they face a two-fold or more increased risk of HIV acquisition. We explored preferences for long-acting PrEP and multipurpose prevention technologies among pregnant and breastfeeding women (PBFW) without HIV. From April to December 2023, we conducted a discrete choice experiment at maternal healthcare clinics in Cape Town, East London (South Africa), and Gaborone (Botswana) including PrEP-experienced and PrEP-naive women. Analysis included site stratification, mixed-effects logistic regression, and latent class modeling. Among 450 pregnant and breastfeeding women (52% pregnant, 47% breastfeeding), vaginally inserted and implanted PrEP was least preferred compared to oral. East London and Gaborone preferred clinic PrEP pick-up and effectiveness, while Cape Town showed indifference for community delivery. Three latent classes emerged: ‘comprehensive delivery seekers’ (43%), ‘physical and physiological prioritisers’ (25%), and ‘vaginal insertion avoiders’ (32%). Future PrEP programs should prioritize user-centered approaches, aligning with user preferences for effective use.

**Supplementary Information:**

The online version contains supplementary material available at 10.1007/s10461-025-04751-6.

## Resumen

El VIH afecta particularmente las mujeres durante el embarazo y el posparto, donde enfrentan un riesgo dos veces o más mayor de contraer el VIH. Las barreras estructurales e individuales obstaculizan el uso eficaz de la profilaxis previa a la exposición (PrEP) diariamente. Exploramos las preferencias por la PrEP de acción prolongada y las tecnologías de prevención multipropósito entre mujeres embarazadas y lactantes sin VIH. Entre abril y diciembre de 2023, evaluamos las preferencias por la PrEP de acción prolongada en un experimento de elección discreta entre mujeres embarazadas, lactantes y posparto que acceden a servicios maternos en el nivel de atención primaria de salud. El estudio incluyó a personas con experiencia previa en el uso de PrEP oral (Ciudad del Cabo, Sudáfrica) y a personas que no habían recibido previamente PrEP (Este de Londres, Sudáfrica y Gaborone, Botswana). El experimento de elección discreta se desarrolló a través de entrevistas cualitativas y discusiones de grupos focales. El análisis incluyó caracterización demográfica, estratificación de sitios, regresión logística de efectos mixtos y modelado de clases latentes. Se encuestaron 450 mujeres embarazadas y lactantes (52% embarazadas, 47% lactantes). Las mujeres estaban fuertemente en contra de la PrEP implantada y de inserción vaginal en comparación con la PrEP oral. Las preferencias variaron según la población del estudio: la recogida de PrEP en las clínicas fue preferida en el este de Londres y Gaborone, mientras que Ciudad del Cabo mostró más indiferencia hacia la entrega comunitaria. Las mujeres del este de Londres y Gaborone priorizaron la eficacia de la PrEP sobre la frecuencia. Surgieron tres clases latentes: Clase 1, “buscadores de entrega integral” (43%); Clase 2, “priorizadores físicos y fisiológicos” (25%) y Clase 3, “evitantes de la inserción vaginal” (32%). La modalidad de PrEP (acción prolongada), la frecuencia y el lugar de recogida fueron factores importantes en la administración de PrEP. Los futuros programas de PrEP deben priorizar enfoques centrados en el usuario, alineándose con los valores y preferencias del usuario para fomentar un uso eficaz.

## Introduction

South Africa (SA) and Botswana are among the four countries with the highest HIV prevalence globally [1]. In Eastern and Southern Africa, women account for 62% of new HIV infections, with young women (aged 15–24) three times more likely than men to be affected. This region sees 77.5% of 4000 new HIV infections that occur globally each week among this population [2]. The risk of HIV acquisition among women increases by two-fold during pregnancy and postpartum period [3, 4]. Various contributing factors include physiological susceptibilities such as hormonal and immunological changes, coupled with engaging in condomless sex [3]. It is crucial to intensify current preventive initiatives during this vulnerable period to further reduce the continued high rates of maternal and newborn HIV acquisition [3].

National guidelines in SA and Botswana recommend pre-exposure prophylaxis (PrEP) for HIV prevention for pregnant and breastfeeding women (PBFW) [5–8]. Daily oral PrEP is a biomedical modality that is safe and effective when used consistently [5, 9–13]. In SA and Botswana, oral PrEP became an available HIV prevention method at primary healthcare level between 2016 and 2018, with updated guidelines extending to PBFW in 2021 in SA and 2023 in Botswana [6, 7, 14, 15]. Various social and structural barriers, such as healthcare access or system barriers, provider bias, dosing challenges, irregular routines, PrEP misconceptions, lack of support, partner mistrust, and risk perceptions however hinder young women’s access to and uptake of PrEP [16–21].

There is an urgent need for access to novel PrEP products and service modalities addressing unique barriers faced by PBFW, often during transitional periods (e.g., to motherhood, childbirth, and postpartum), and enhancing persistence on PrEP [22–32]. Previously reported product preferences among PBFW include discretion, ease of use, reduced dosing, and minimal physical discomfort [24, 25]. Developed and proposed long-acting PrEP agents include dapivirine vaginal rings [33], long-acting injectables (e.g., cabotegravir or lenacapavir), new oral antiretroviral drugs (e.g., monthly) [34], and implants [35]. Dapivirine vaginal rings and injectable cabotegravir for PrEP were registered in SA in 2022. Dapivirine rings are not yet approved for use among pregnant women [36–39]. In Botswana, the dapivrine ring is not yet approved. Injectable cabotegravir is not contraindicated for PBFW in SA nor Botswana, and implementation studies have started in SA, and safety trials in postpartum women are ongoing in Botswana in 2024 [7]. Additionally, women have shown a desire for multipurpose prevention technologies incorporating both HIV and contraception needs [25, 40–42].

Assessing users’ preferences and decision-making through discrete choice experiments (DCE), based on economic theory, is an effective way to understand and address existing barriers for PrEP by aligning future PrEP alternatives and delivery options with individual values and preferences [43–45]. Women of childbearing age continue to be underrepresented in clinical trials due to safety and fertility concerns, resulting in limited access and safety data, along with provider initiation hesitation [46, 47]. As a result, DCEs are particularly useful for assessing product preferences among this population to inform novel product design and service modality strategies [8, 44, 48–52].

Given the high incidence of HIV in PBFW in southern Africa and the increasing availability of preventive options, we conducted a DCE among PBFW accessing maternal services in SA and Botswana to determine preferences for long-acting PrEP and multipurpose prevention technologies.

## Methods

PrEP-Choice was a cross-sectional study employing a mixed-methods approach to assess attitudes and preferences for long-acting PrEP service delivery modalities among PBFW without HIV. Women were purposively sampled during routine antenatal and postnatal services from collaborating public or community healthcare facilities. We report findings derived from behavioral survey responses from PBFW with prior PrEP experience. We explored variations in DCE responses for PrEP delivery preferences based on PrEP exposure experience, physiological period, and geographical location.

### Study Population and Sampling

Participants were recruited between April and December 2023 from three southern African sites: (1) Cape Town, SA; (2) East London, SA; and (3) Gaborone, Botswana, with antenatal care HIV prevalence estimated from previous studies at 20.3%, 29%, and 17%, respectively [53–55]. Collaborating healthcare facilities in East London were defined to cater to peri-urban and rural areas, whereas Cape Town and Gaborone were predominantly characterized as urban. Cape Town has a larger population of oral PrEP-experienced women through well-established implementation projects such as Fast-PrEP and PrEP-PP [48]. All three study sites have established relationships with local health districts and have worked extensively on clinical and implementation science studies [12, 28, 29, 56, 57].

Trained researchers approached PBFW using a standardized script for screening. Overall eligibility included: ≥18 years of age; documented HIV-negative test at most recent test (per national protocol for routine antenatal and postnatal care); any gestational age for pregnant women and women breastfeeding up to 3-months postpartum, and ability and willingness to provide informed consent. A sample size of at least 150 participants per site would ensure sufficient power to detect 15–20% preference differences for PrEP delivery by site. The sampling aimed to achieve an equal 1:1 ratio of pregnant and breastfeeding women, with each perinatal group including between 65 and 85 participants per site. In Cape Town, PBFW with prior or current daily oral PrEP experience (PrEP-experienced), including PBFW who had no prior experience taking PrEP (PrEP-naïve), were recruited, while only PrEP-naïve PBFW were enrolled in East London and Gaborone. This approach facilitated a diverse evaluation of long-acting PrEP preferences, considering contextual factors by site while considering PrEP roll-out phases, geographic locations, and different physiological periods. Written informed consent, which was presented in English, Setswana, or IsiXhosa to cater to participants’ language preferences, was obtained immediately after eligibility screening. PBFW expressing interest in oral PrEP initiation were referred per standard of care at collaborating healthcare facilities.

**Table 1:** PrEP choice discrete choice experiment attributes and levels

Attribute	Level 1	Level 2	Level 3	Level 4
Clinic visit (and product refill)	Refill every month	Refill every 3 months	Refill every 6 months	–
Discomfort with PrEP use incl. side effects	Moderate discomfort or side effects (will feel headaches, fatigue, nausea or vomiting, or site injection pain, but it goes away)	Mild discomfort or side effects (may feel headaches, fatigue, nausea or vomiting or site injection pain, but it goes away)	No discomfort or side effects (can’t feel it nor tell you are using it)	–
Types of PrEP product	Oral pill	Vaginal inserted	Injection in arm or buttock	Implant in arm
Combination of prevention methods	HIV prevention only	HIV and STI prevention	HIV and pregnancy prevention	HIV, STIs and pregnancy prevention
Pick-up location	Government clinic pick-up	Mobile community delivery (e.g., community delivery point or mobile van)	Private pharmacy pick-up (e.g. Clicks)	–
Effectiveness and duration of protection	Very effective (75–90%) and take more frequently e.g., daily	Very effective (75–90%) and take less frequently e.g., monthly	Moderately effective (35–50%) and take more frequently e.g., daily	Moderately effective (35–50%) and take less frequently e.g., monthly

### Discrete Choice Experiment Survey Design

The final DCE design included six attributes (Table 1), informed by in-depth interviews and focus group discussions (qualitative findings are under consideration in a separate manuscript) [58]. The attribute definitions and levels were refined through findings and investigator meetings.

Choice sets were designed using dcreate in Stata18 (StataCorp LLC College Station, Texas), using a modified Federov algorithm to maximize the D-efficiency of the design based on the covariance matrix of the conditional logit model [59–62]. A binary, unlabeled, fractional factorial design with 40 choice sets was generated, and the design was divided into four versions so that each participant only answered 10 of the 40 choice sets. The design did not include an opt-out option (encouraging participants to either select Option A or B) to maximize the amount of information collected on participant preference structures. The final tool presented choice sets using images for each attribute level including labels, which were refined following a pilot among a small number of participants. All attributes and descriptions were translated and available to participants in English, isiXhosa (SA) or Setswana (Botswana).

### Survey Administration

Prior to DCE administration, participants responded to sociodemographic survey questions, including questions about the perinatal stage, partner HIV infection status, distance to clinic, and family planning methods. Participants from Cape Town were asked additional behavioral questions about their daily oral PrEP experiences, including side effects, adherence, and barriers, and in comparison, their attitudes toward long-acting injectables and multipurpose technologies were measured. Trained research assistants read each attribute aloud to participants using a standardized guide, showing images of each attribute level, and confirming participants’ understanding of each attribute before explaining the next set of attribute levels.

**Fig. 1:**
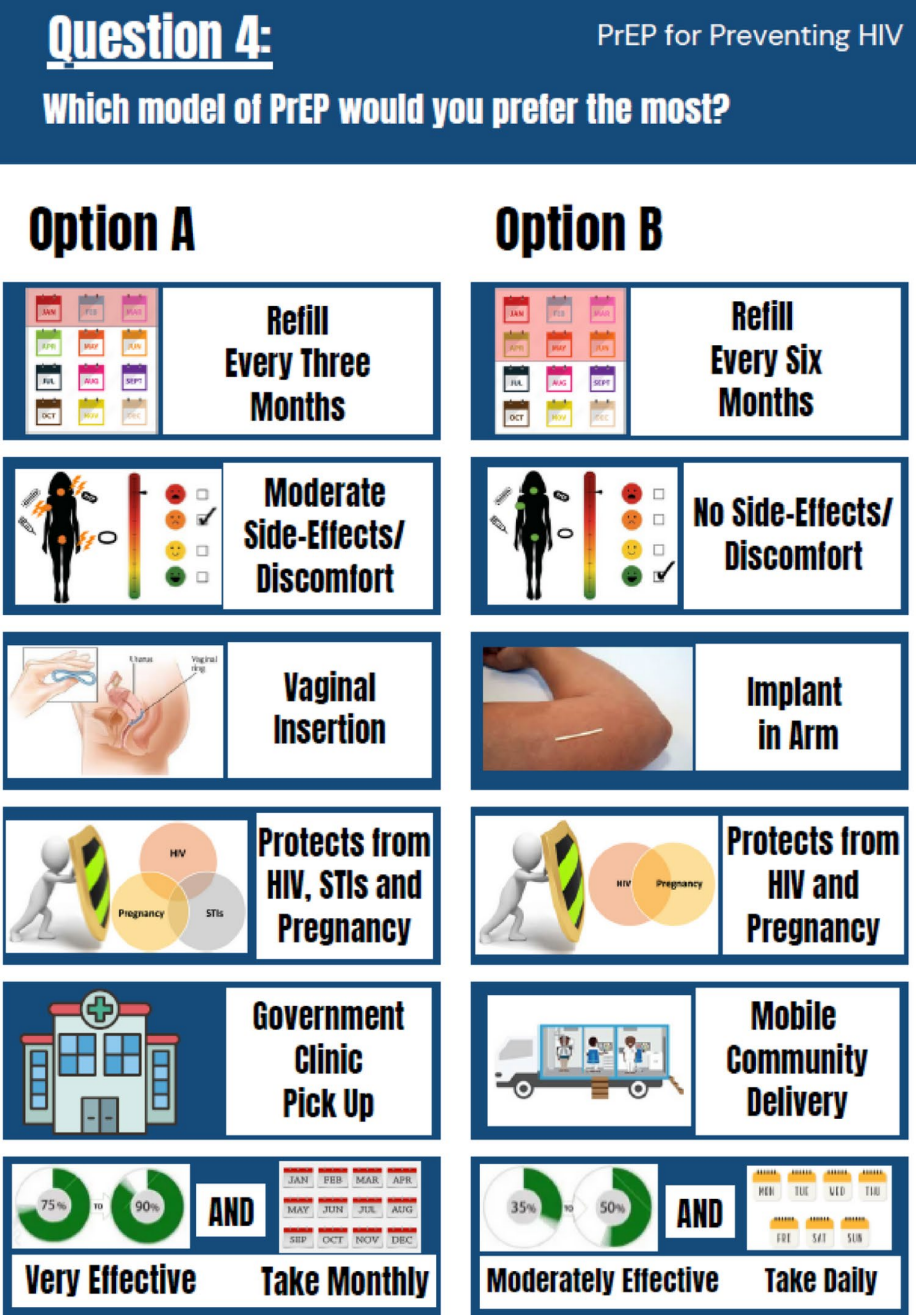
Example of a PrEP Choice discrete choice experience task presented to participants

Participants were presented with an allocated version according to a predetermined list to ensure a balance of each version. For every question, participants were asked, “Which model of PrEP would you prefer the most?” (Fig. 1). This prompted participants to choose between two theoretical PrEP delivery packages (Option A or B) considering each attribute and level possibility. Surveys were research assistant-administered with responses captured on REDCap and took approximately 30–45 min to complete.

### Data Analysis

Participant demographic characteristics were analysed and are presented as descriptive frequencies using STATA v.18 (Stata Corporation, College Station, Texas). Data were further stratified by site to compare the preferences of PBFW among oral PrEP users in Cape Town to those who were PrEP naïve in East London and Gaborone. We compared preferences between women who were pregnant and breastfeeding/postpartum women.

The primary model used for analysis was the mixed effects binary logistic regression model. Likelihood ratio chi2 tests and log-likelihood statistics were used for all the mixed effects models representing the standard errors, z-statistics, and 95%Cis. The standard deviation estimates for the main and stratified models have been included as Supplementary information. Attribute levels were dummy-coded, and mean utility coefficients for each level were estimated using predetermined reference levels with 1000 Halton draws for simulations. The preference strength for an attribute (positive or negative) was shown through the value of the coefficient in relation to its reference level. These findings further reveal whether these characteristics influenced a participant’s decision to choose Option A or B. Standard deviation estimates were generated to show the magnitude and significance of preference heterogeneity within the sample to indicate where further investigation of divergence in preferences might be warranted. For 95% Cis that overlap with zero, this was an indication that participants were more indifferent between the characteristic in question and the reference level. Stratified models were used to analyze differences by study site (Cape Town, East London, and Gaborone), age (women ≤ 24 years versus women ≥ 25 years old), and perinatal period (pregnant versus postpartum/breastfeeding).

Latent class models were used to further explore preference heterogeneity [63]. Latent class models assume that there are groups of participants within the sample who have similar preference structures even when group membership is not known beforehand. These models estimate the probability of class membership given the number of classes prespecified by the analyst based on individual-specific preference weights and then use conditional logit models to estimate coefficients for each of the attribute levels in each class. This type of analysis can inform tailored interventions and optimize strategies by uncovering sub-group preferences and behaviors that are otherwise complex or not evident and further accommodate categorical data and measurement error. In this analysis, four-class, three-class, and two-class models were estimated, and model statistics and model estimates were compared to select the most appropriate model for presentation (Table 2). The Akaike information criterion and Bayesian information criterion statistics are commonly used measures of model fit but produce conflicting results because of how they are calculated. Mean probability of class membership was high in all models. We selected the three-class model for presentation and classes were assigned qualitative labels—developed by the researchers—to describe the main preference structures in each group.

**Table 2:** Latent class model fit statistics for two-, three-, and four-class models in PrEP-Choice discrete choice experiment (*n* = 450 pregnant and breastfeeding women)

	Number of observations	Log-likelihood	AIC	BIC criterion	Mean probability of class membership
2-class model	8100	−2374.3348	4810.6695	4938.0562	0.89
3-class model	8100	−2329.7318	4753.4637	4946.5983	0.85
4-class model	8100	−2296.2721	4718.5443	4977.4269	0.84

AIC = Akaike information criterion; BIC = Bayesian information

### Ethics

This study obtained approval from Faculty of Health Sciences Human Research Ethics Committee at the University of Cape Town (Ref: 619/2022) and Botswana Health Research Development Committee (Ref: HRDC #0098). Participants were reimbursed for their time.

## Results

A total of 450 PBFW participated in the study (52% pregnant and 47% breastfeeding). The median age of participants overall was 26 years (IQR 22–31). Participant characteristics for each site are shown in Table 3. In Gaborone, participants were more likely to be unmarried/not living with their partner (62%) than married/cohabiting in East London and Cape Town (51–53%). More participants in East London reported having no partner (*n* = 21/150,14%). Of all participants, 20% (*n* = 88) did not know the HIV status of their partner, and more participants reported a partner living with HIV in Cape Town (9%).

**Table 3:** Characteristics of participants in the discrete choice PrEP experiment stratified by site (*n* = 450)

Characteristics		Cape Town (*n* = 150)	East London (*n* = 150)	Gaborone (*n* = 150)	Total (*n* = 450)
Age in years (median, IQR)		26 (22–32)	27 (22–32)	25 (22–29)	26 (22–31)
Pregnant or postpartum/breastfeeding	Pregnant	76 (51%)	75 (50%)	85 (56%)	236 (52%)
	Postpartum	74 (49%)	75 (50%)	66 (44%)	215 (47%)
Relationship status	Married/living with partner	80 (53%)	77 (51%)	54 (36%)	211 (47%)
	Unmarried/not living with partner	67 (45%)	52 (35%)	93 (62%)	212 (47%)
	No partner	3 (2%)	21 (14%)	4 (3%)	28 (6%)
Partner HIV status	Don’t know	43 (29%)	25 (17%)	20 (13%)	88 (20%)
	HIV Negative	91 (61%)	100 (67%)	124 (82%)	315 (70%)
	Living with HIV	13 (9%)	4 (3%)	1 (1%)	18 (4%)
	Refused	0 (0%)	0 (0%)	2 (1%)	2 (0%)

Among 150 participants in Cape Town, 76 pregnant women and 74 postpartum women participated in a behavioral survey to determine their oral PrEP experiences and preferences (Supplemental Table 1). They had been using PrEP for a median of 84 days (40–152). The majority (> 90%) reported previous use of injectable contraceptives and/or condoms. All participants emphasized HIV prevention as the most liked characteristic of PrEP over other features. Compared with 20% of breastfeeding women, 32% pregnant women disliked side effects of PrEP. Similarly, more pregnant women (16%) disliked daily dosing than breastfeeding women (4%). One-quarter of pregnant women expressed fears of side effects. Most participants (> 95%) reported no shame about PrEP or concerns regarding their partner finding out.

### Main Effects Across all Sites

**Fig. 2:**
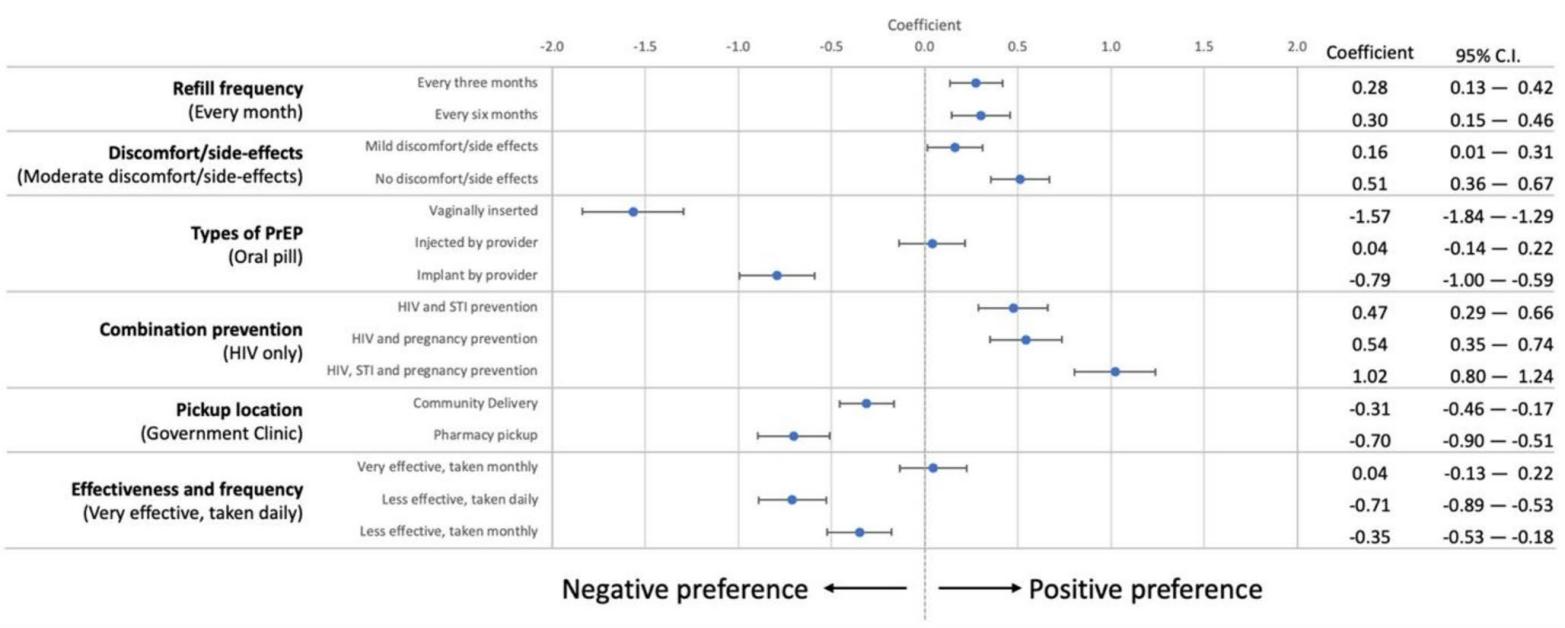
Mean estimates of PrEP preferences for all study participants (*n* = 450 PBFW)

Almost all attribute levels had significant coefficients, demonstrating where preferences for certain attribute characteristics most diverged (Supplemental Table 2). Figure 2 depicts PBFW’s PrEP attribute preferences across all three settings (Cape Town, East London, and Gaborone). Results demonstrate that participants preferred not to receive vaginally inserted (coefficient −1.57, 95% CI = −1.84, −1.29) or implanted PrEP (−0.79, 95% CI = −1.00, −0.59) versus oral PrEP. Similarly, participants strongly favored combination prevention, including HIV, STIs, and pregnancy (1.02, 95% CI =  0.80, 1.24), with a notable preference for combinations beyond HIV prevention alone. Notably, community delivery was less preferred (−0.31, 95% CI = −0.46, −0.17), while private pharmacy collection was least preferred compared to government clinic pick-up (−0.70, 95% CI = −0.90, −0.51). There was no significant difference in preferences for frequency of use when PrEP was more effective, but when it was less effective, participants showed a preference for less frequent dosing. Participants favored a method that had no side effects or discomfort (0.51, 95% CI = 0.36, 0.67) compared to moderate side effects or discomfort, although this preference was not as strong compared to other characteristics. While there was a slight preference for less frequent refills compared to monthly refills, no discernible difference was found between 3- or 6-month refill intervals (0.28; 95% CI = 0.13, 0.42 versus 0.30, 95% CI = 0.15, 0.46).

### Main Effects by Site

**Fig. 3:**
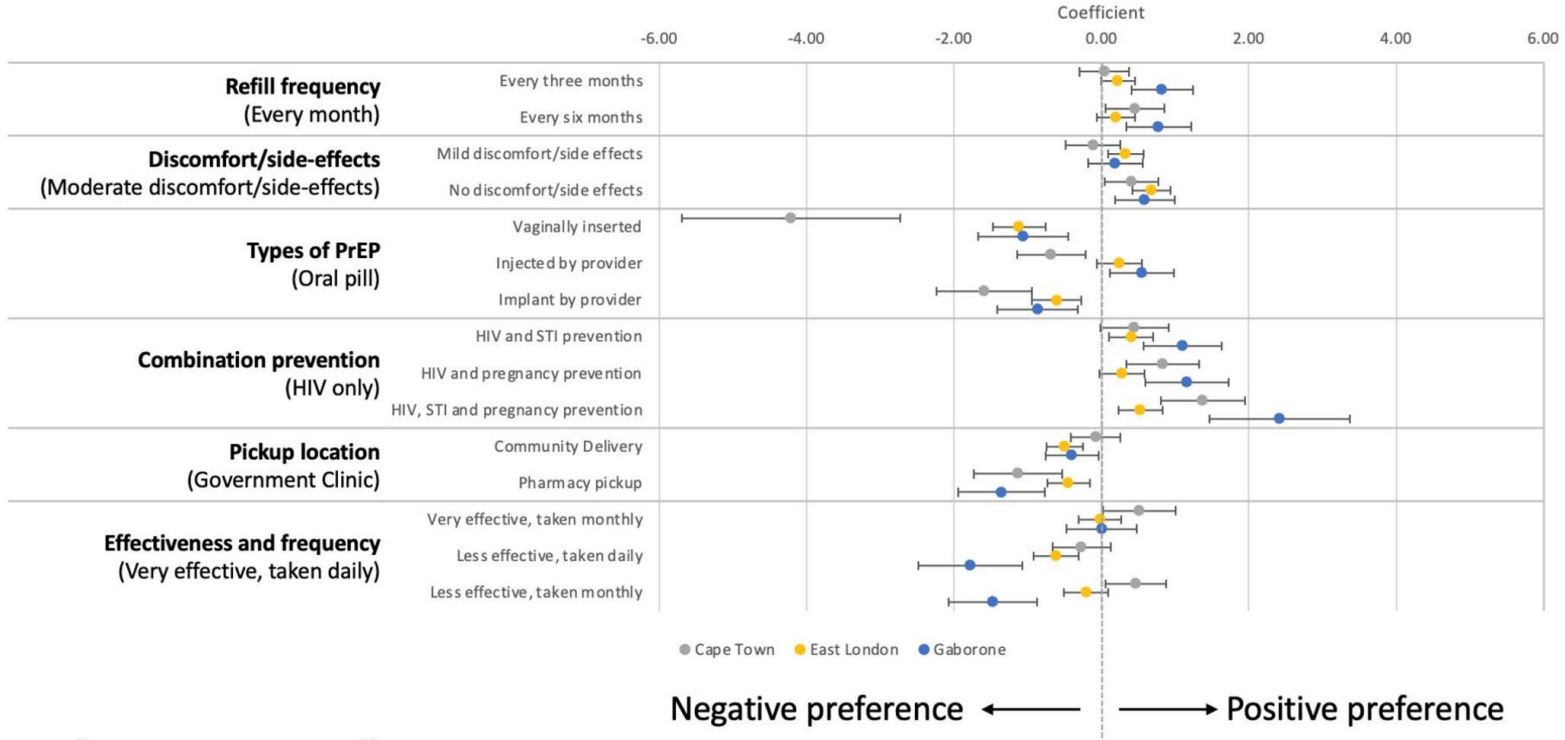
PrEP delivery preferences by study site (Cape Town, East London, and Gaborone)

Figure 3 depicts the preferences by site, including their mean coefficients, and standard deviation estimates to show population heterogeneity (Supplementary Table 3). Oral PrEP was strongly preferred to vaginally inserted or implanted PrEP. However, combination prevention methods were preferred in Cape Town (1.37, 95% CI = 0.80, 1.95) and East London (0.53, 95% CI = 0.23, 0.83) compared to methods that prevent only HIV, with the strongest preference observed in Gaborone (2.42, 95% CI = 1.47, 3.37). In East London, the difference in preference for type of combination prevention was less pronounced. Conversely, East London participants were indifferent to injectable or oral PrEP (0.24, 95% CI = −0.07, 0.55), while in Gaborone, a preference for injectable PrEP was shown (0.55, 95% CI = 0.12, 0.98). Clinic pick-up for PrEP was favored in East London and Gaborone, with a negative preference between pharmacy pick-up (−0.45, 95% CI = −0.74, −0.16) and community pick-up (−0.50, 95% CI = −0.75, −0.25) in East London compared to Gaborone (−1.36, 95% CI = −1.95, −0.77). There was no difference in choice in Cape Town between clinic pick-up and community delivery (−0.08, 95% CI = −0.42, 0.26). Effectiveness of PrEP was prioritized over frequency of use in East London and Gaborone, while in Cape Town, dosing frequency had greater significance than effectiveness. Discomfort or side effects were less important in Cape Town and Gaborone, but women in East London significantly preferred no mild discomfort/side-effects (0.68, 95% CI = 0.42, 0.94) compared to moderate effects. Although PBFW in Cape Town and East London slightly preferred infrequent refills over monthly refills, the difference was not significant. In Gaborone, less frequent refills were preferred, with no difference between 3-month (0.82, 95% CI = 0.41, 1.24) and 6-month intervals (0.77, 95% CI = 0.33, 1.21).

### Interactions by Pregnancy or Postpartum Status and Maternal Age

Participants across different perinatal periods and age groups exhibited similar PrEP delivery preferences (Supplemental Figs. 1 and 2, Supplementary Tables 4 and 5). Pregnant women were less likely to opt for moderately effective, frequently used PrEP (−0.71, 95% CI = −0.89, −0.53) in relation to highly effective daily PrEP. Additionally, younger women (< 25 years) were more hesitant toward vaginal insertion versus orally administered PrEP (−2.01, 95% CI = −2.57, −1.45).

### Latent Class Analysis

**Fig. 4:**
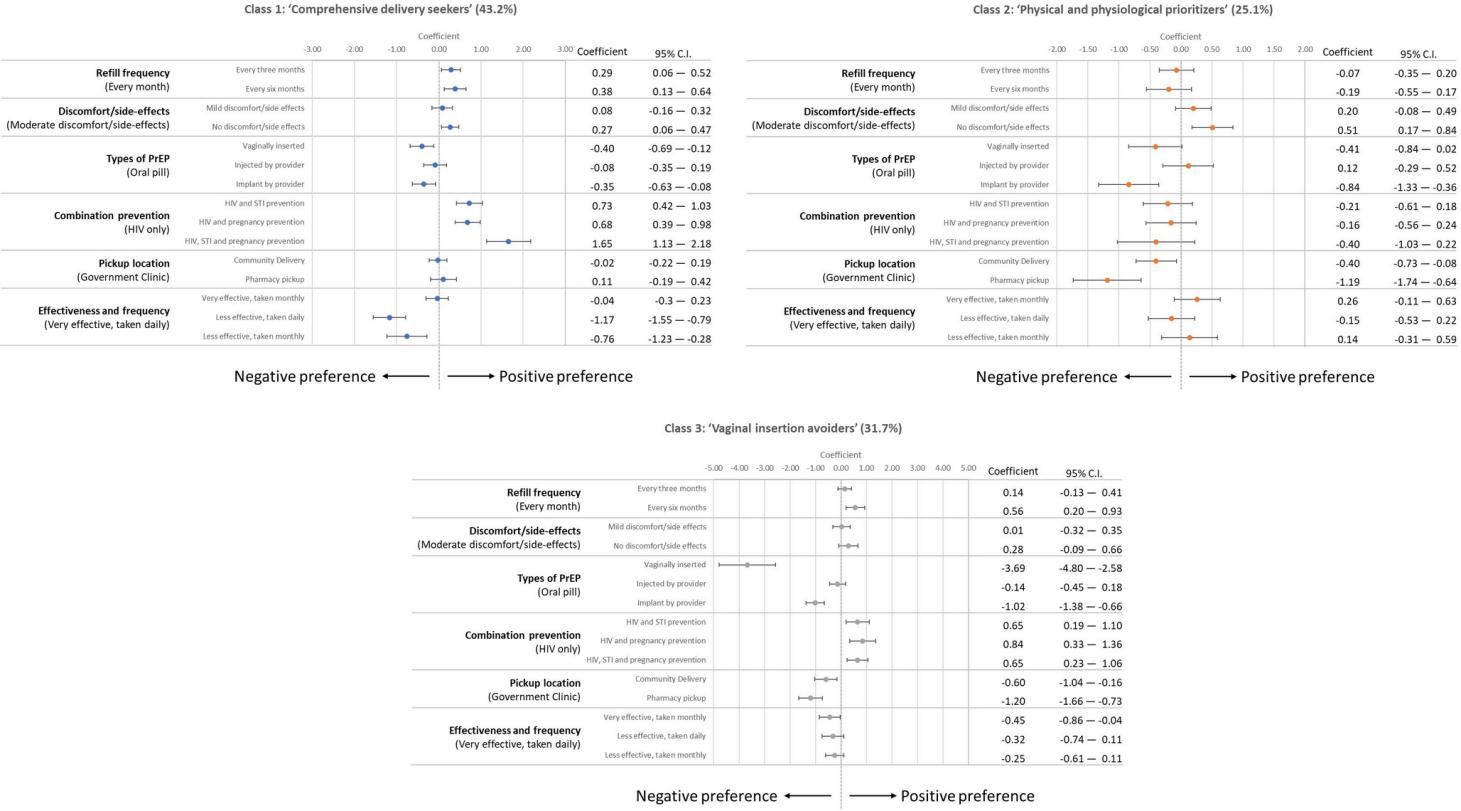
Latent class model analysis identifying three classes that effectively describe PBFW (*n* = 450) PrEP delivery preference

Latent class model analysis identified three classes that effectively described PBFW’s PrEP delivery preferences (Fig. 4). The largest group (43%) fell into Class 1, where PBFW prioritized combination prevention and PrEP dosing frequency: ‘comprehensive delivery seekers’. Class 3 was the second largest group (32%), where preferences were primarily driven by avoidance of vaginal insertion, and participants prioritized combination prevention, pickup location, and dislike of implants. This group was assigned a qualitative label comprising ‘vaginal insertion avoiders’. Last, 25% of participants fell into Class 2, favoring physical and physiological aspects of PrEP, such as pickup locations and side effects, as well as showing a strong rejection of implants: ‘physical and physiological prioritizers’.

In Class 1, ‘comprehensive delivery seekers’, women strongly favored combination prevention over HIV-only prevention, particularly for STIs, HIV, and pregnancy prevention (1.65, 95% CI = 1.13, 2.18). They also had a significant aversion to less effective PrEP, regardless of dosing frequency (−1.17, 95% CI = −1.55, −0.79 and −0.76, 95% CI = −1.23, −0.28). Compared to other groups, there were less pronounced preferences for vaginally inserted (−0.40, 95% CI = −0.69, −0.12) or implanted (−0.35, 95% CI = −0.63, −0.08) PrEP than for oral PrEP. Similarly, there was no strong preference for refill frequency, pick-up location, nor discomfort/side effects.

In Class 2, ‘physical and physiological prioritizers’, women prioritized government clinic pickup over other options and particularly strongly opposed pharmacy pick-up (−1.19, 95% CI = −1.74, −0.64). This group showed a negative preference for PrEP implants (−0.84, 95% CI = −1.33, −0.36) compared to oral PrEP, with no difference in preference for injections (0.12, 95% CI = −0.29, 0.52) nor vaginal insertion (−0.41, 95% CI = −0.84, 0.02). They strongly preferred no side-effects/discomfort (0.51, 95% CI = 0.17, 0.84) opposed to moderate side effects. Although they were indifferent to characteristics relating to refill frequency, they showed no real preference for combination prevention options or dosing effectiveness/frequency compared to their relevant baseline characteristics.

PBFW in the Class 3 group, ‘vaginal insertion avoiders’, were strongly motivated by avoiding vaginal insertion (−3.69, 95% CI = −4.80, −2.58). Unlike Class 2, they showed a positive preference for any combination prevention over HIV-only prevention. Additionally, although less pronounced than Class 2, they displayed a negative association with community delivery (−0.60, 95% CI = −1.04, −0.16) or pharmacy pick-up (−1.20, 95% CI = −1.66, −0.73) compared to government clinic pick-up. Similarly, attributes relating to refill frequency and dosing effectiveness concerning dosing frequency were not of significant concern.

## Discussion

Our study revealed that choice of PrEP method, frequency of prescription, and delivery choice were key preferences among PBFW in SA and Botswana. Overall, PBFW preferred less frequent prescription refills and clinic visits (> 3 months). Most preferred a method with no or only mild discomfort or side effects. Injectable PrEP is commonly preferred in PBFW in SA, where contraceptive methods are commonly injectable [25]. There was a negative preference for use of vaginal rings, including fear of associated discomfort or pain. PBFW instead preferred familiarity or convenience of oral pills. They were more concerned with health system barriers, including the frequency of PrEP refills versus effectiveness. PBFW who were PrEP naïve had negative preferences for community delivery, perhaps due to experienced or anticipated stigma. Similarly, PBFW have experience accessing care at government clinics and may prefer the integration of services such as antenatal care or family planning [64]. In Kenya, pregnant women favored clinics over pharmacies for HIV prevention due to lower perceived costs and encouragement for perinatal care at primary healthcare facilities [65]. However, community-based and differentiated PrEP services have increased uptake among AGYW [66]. In Cape Town, PBFW women showed similar preferences for community delivery and public clinics potentially due to increased service models in this area, highlighting the importance of the diversification of services that cater to diverse user needs especially during transitional periods. In this study, collective preferences among PBFW were investigated through latent class analysis, which revealed ‘comprehensive delivery’, ‘vaginal insertion’, and ‘physical and physiological’ priorities. The findings highlight distinct sub-group preferences and heterogeneity based on attribute types. While some groups prioritize combination prevention, revealing a boarder need for sexual and reproductive health and high efficacy (Class 1), others place greater emphasis on accessibility or physical comfort (Class 2). Concerns about discomfort or invasiveness with specific PrEP methods, such as vaginal insertion (Classes 1 and 3) and implants, suggest that unfamiliarity with alternative formats could dissuade use, underscoring the need for demonstration programs and a range of well-tolerated options by end-user. Additionally, refill frequency was less heavily weighted across groups, indicating flexibility in this attribute in light of other future long-acting PrEP attributes. These findings emphasize that designing equitable and effective HIV prevention strategies for PBFW requires carefully balancing diverse user needs. It is important to note that these findings reflect preferences for PBFW accessing services from public clinics.

Given the recent regulatory approval of new PrEP modalities in SA and Botswana, it is essential to address implementation strategies and counseling around choice to ensure maximum effectiveness. High efficacy of long-acting injectable cabotegravir and modest efficacy of the dapivirine vaginal ring make use of new PrEP modalities for PBFW at risk of HIV an urgent ethical and research priority [67]. DCE outcomes based on preferences are a critical starting point for clinical work and programmatic studies among this population, but there is no risk for PBFW. While safety and efficacy data accumulate for PBFW and their infants [67, 68], there is an urgent need for implementation studies that focus on how to provide PrEP choices among PBFW, particularly around a scalable implementation strategy that assists PBFW in choosing PrEP methods that correspond to their needs and values to maximize its effectiveness.

Initial results from the HPTN-084 open-label extension demonstrated that in 1000 women, 78% chose to start or continue CAB-LA, and 68% of 233 pregnant women chose to take CAB-LA. Product choice was influenced by personal preference for product attributes, social context, and risk behaviors; participants expressed limited decisional conflict [69]. In the MTN-034/REACH crossover trial among nonpregnant African adolescent girls and young women, adherence was high in those who were given the choice between oral PrEP and dapivirine ring; 57% of visits in women on oral PrEP, and the ring had high adherence validated with objective measures [39]. To date, PBFW in Africa have not had a choice of highly effective prevention modalities; this choice is critical to their decision-making process, which may improve the effectiveness of PrEP products by encouraging longer-term persistence and adherence [70].

Many PrEP findings have also shown that one-size-fits-all implementation models do not accommodate all needs and barriers among end-users, resulting in low uptake despite availability [71, 72]. However, current health systems may not be able to accommodate all tailored service delivery models at the individual level. This means that if health systems can only target or implement one model, it should work well for the main target population, as reflected by the preference findings. If multiple service delivery models are feasible, latent class analysis can reveal where preferences overlap and where they most diverge for delivery models.

The hypothetical nature of the experimental design is both a strength and a weakness of this study. Results relied on a stated preference approach to understanding participant choice, and choices made by participants may not perfectly align with actual choices made in real-world settings. The smallest latent class (< 100 participants) limits the detection of statistically significant trade-offs. Variability in PrEP exposure across study sites, influenced by existing delivery interventions, enhanced sample diversity among PBFW that could occur in the real world but also introduced differences in preferences shaped by experience versus perceptions of PrEP. Further, this study focused primarily on delivery characteristics and PrEP products. Future research is required to better understand how important social and structural factors are in determining preferences and how these factors are linked to the service delivery model and product design in driving demand for PrEP overall.

## Conclusion

PrEP modality, frequency, and pickup location are crucial in PrEP delivery. Recognized for its practical and ethical value, patient-centered care emphasizes involving patients directly. Shared decision-making counseling between providers and clients may enhance patient-centered care quality and communication. These approaches further enhance alignment with PBFW’s values and preferences to foster effective use. Furthermore, newer modalities, including long-acting pills, injections, or implants, have the potential to significantly reduce HIV acquisition and vertical transmission.

## Electronic Supplementary Material

Below is the link to the electronic supplementary material.

Supplementary Material 1

Supplementary Material 2

Supplementary Material 3

Supplementary Material 4

Supplementary Material 5

Supplementary Material 6

Supplementary Material 7

## Data Availability

The data that support the findings of this study are available from the corresponding author upon reasonable request including a data request form.
