# Supplementary material for: Preference for Service Delivery for Long-Acting Pre-exposure Prophylaxis for HIV Infection Among Pregnant and Breastfeeding Women in South Africa and Botswana
Source: AIDS Behav. 2025 May 21;29(9):2963–75. doi: 10.1007/s10461-025-04751-6 (PMC12432069; doi:10.1007/s10461-025-04751-6)
Supplement: Supplementary file 1 — Supplementary Material 1 [file 10461_2025_4751_MOESM1_ESM.pdf]

Supplementary Information

Supplemental Table 1. Demographics and PrEP use among PBFW in the PrEP Choice DCE Study, Cape Town, SA (n=150 women)

**Demographics**	**Total (n=150)**	**Pregnant (n=76)**	**Postpartum (n=74)**
Age (median, IQR)	26 years (22-32)	28 years (22-33)	25.5 (21-30)
Gestational age (weeks, median, IQR)		31.5 weeks (24-35)	
Postpartum stage (weeks, median, IQR)	56 weeks (28-88)		64 weeks (34-109)
Currently married or cohabiting	80 (53%)	39 (51%)	41 (55%)
Partner HIV serostatus			
Living with HIV	13 (9%)	7 (9%)	6 (8%)
Unknown status	43 (29%)	21 (28%)	22 (30%)
Distance from home to clinic			
<5 kilometers	36 (24%)	22 (29%)	14 (19%)
5-10 kilometers	102 (68%)	46 (61%)	56 (76%)
10-30 kilometers	10 (6%)	6 (8%)	4 (5%)
Time on oral PrEP (median, IQR)	84 days (40-152)	75 days (31, 132)	84 days (41-152)
Contraceptive use in past			
Oral contraceptive pill	47 (31%)	22 (29%)	25 (34%)
Contraceptive injection	140 (93%)	72 (95%)	68 (92%)
Condom	136 (91%)	65 (86%)	71 (96%)
Common likes about oral PrEP			
HIV prevention	150 (100%)	76 (100%)	74 (100%)
Ease of use	57 (38%)	32 (42%)	25 (34%)

Doesn’t interrupt sex	58 (39%)	33 (43%)	26 (35%)
Common dislikes about oral PrEP			
Side effects	39 (26%)	24 (32%)	15 (20%)
Dislike pills, size of pills	15 (10%)	12 (16%)	3 (4%)
No dislikes	95 (63%)	42 (55%)	53 (72%)
Feels ashamed of PrEP			
Strongly agree or agree	2 (1%)	1 (1%)	1 (1%)
Strongly disagree or disagree	148 (99%)	75 (99%)	73 (99%)
Fear partner may determine			
Strongly agree or agree	4 (3%)	2 (3%)	2 (3%)
Strongly disagree or disagree	146 (97%)	74 (97%)	72 (97%)
Worried about side effects of PrEP			
Strongly agree or agree	27 (17%)	18 (24%)	14 (19%)
Strongly disagree or disagree	123 (82%)	58 (76%)	60 (81%)
