# Supplementary material for: Preference for Service Delivery for Long-Acting Pre-exposure Prophylaxis for HIV Infection Among Pregnant and Breastfeeding Women in South Africa and Botswana
Source: AIDS Behav. 2025 May 21;29(9):2963–75. doi: 10.1007/s10461-025-04751-6 (PMC12432069; doi:10.1007/s10461-025-04751-6)
Supplement: Supplementary file 2 — Supplementary Material 2 [file 10461_2025_4751_MOESM2_ESM.pdf]

Supplementary Information

Supplemental Table 2. Coefficients, p-values and 95% confidence intervals derived from the PrEP-CHOICE discrete choice experiment (n=450 pregnant and breastfeeding women)

Attribute	Level	Coefficient	P-value	95% confidence interval	
Refill frequency (Every month)	Every three months	-0.03	0.855	-0.38	0.32
	Every six months**	0.52	**<0.001**	0.24	0.79
Discomfort/side-effects (Moderate)	Mild discomfort/side effects	-0.18	0.442	-0.64	0.28
	No discomfort/side effects	0.14	0.525	-0.29	0.57
Types of PrEP (Oral pill)	Vaginally inserted**	1.58	**<0.001**	1.27	1.89
	Injected by provider**	0.94	**<0.001**	0.65	1.22
	Implant by provider**	0.96	**<0.001**	0.67	1.25
Combination prevention (HIV only)	HIV and STI prevention	0.03	0.937	-0.64	0.70
	HIV and pregnancy prevention	0.15	0.640	-0.48	0.78
	HIV, STI and pregnancy prevention**	0.77	**<0.001**	0.48	1.07
Pickup location (Government Clinic)	Community Delivery*	0.35	**0.023**	0.05	0.65
	Pharmacy pickup**	1.00	**<0.001**	0.76	1.24
Effectiveness and frequency (Very effective, taken daily)	Very effective, taken monthly**	-0.54	**0.003**	-0.89	-0.18
	Less effective, taken daily**	-0.53	**0.005**	-0.90	-0.16
	Less effective, taken monthly**	0.50	**0.008**	0.13	0.87

Bold p<0.5
