# Supplementary material for: Preference for Service Delivery for Long-Acting Pre-exposure Prophylaxis for HIV Infection Among Pregnant and Breastfeeding Women in South Africa and Botswana
Source: AIDS Behav. 2025 May 21;29(9):2963–75. doi: 10.1007/s10461-025-04751-6 (PMC12432069; doi:10.1007/s10461-025-04751-6)
Supplement: Supplementary file 3 — Supplementary Material 3 [file 10461_2025_4751_MOESM3_ESM.pdf]

Supplementary Information

Supplementary Figure 1. Results: The main discrete choice experiment effects on pregnant versus postpartum women

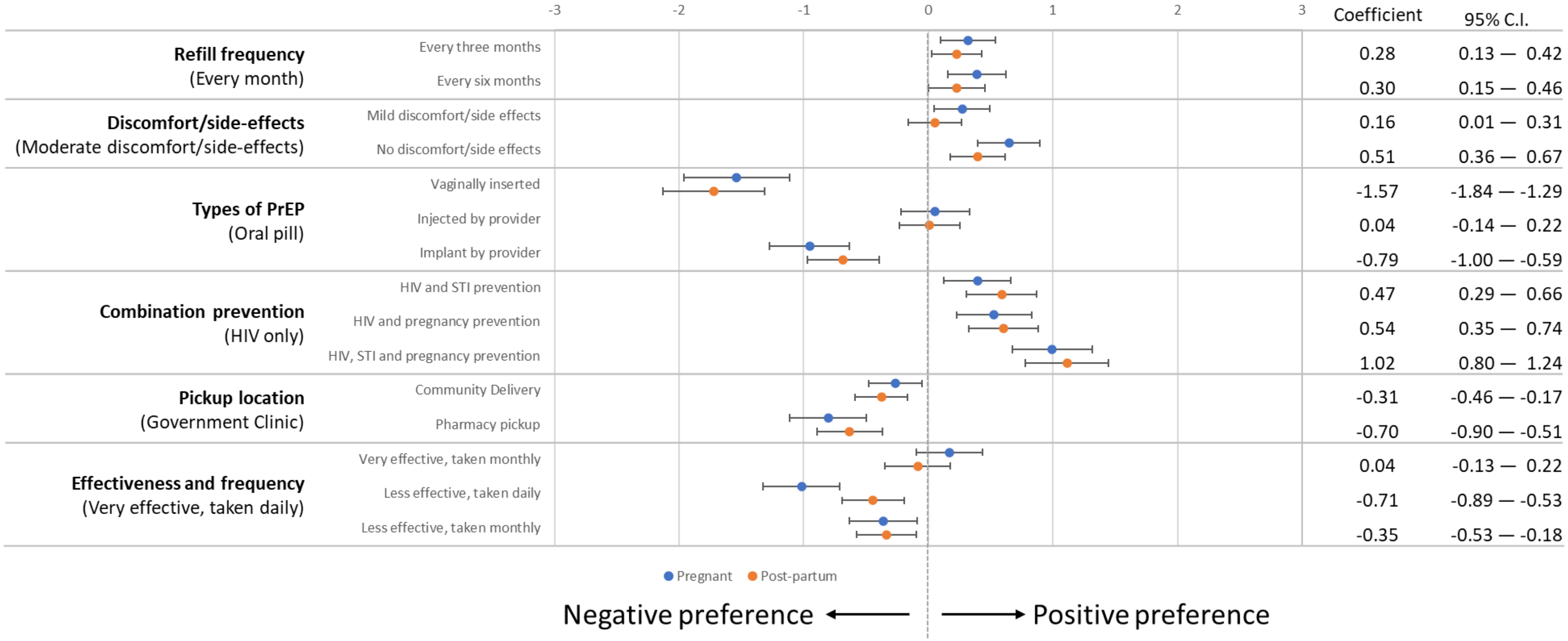

Attribute (reference)	Level	Coefficient	95% C.I.
Refill frequency (Every month)	Every three months	0.28	0.13 — 0.42
	Every six months	0.30	0.15 — 0.46
Discomfort/side-effects (Moderate discomfort/side-effects)	Mild discomfort/side effects	0.16	0.01 — 0.31
	No discomfort/side effects	0.51	0.36 — 0.67
Types of PrEP (Oral pill)	Vaginally inserted	-1.57	-1.84 — -1.29
	Injected by provider	0.04	-0.14 — 0.22
	Implant by provider	-0.79	-1.00 — -0.59
Combination prevention (HIV only)	HIV and STI prevention	0.47	0.29 — 0.66
	HIV and pregnancy prevention	0.54	0.35 — 0.74
	HIV, STI and pregnancy prevention	1.02	0.80 — 1.24
Pickup location (Government Clinic)	Community Delivery	-0.31	-0.46 — -0.17
	Pharmacy pickup	-0.70	-0.90 — -0.51
Effectiveness and frequency (Very effective, taken daily)	Very effective, taken monthly	0.04	-0.13 — 0.22
	Less effective, taken daily	-0.71	-0.89 — -0.53
	Less effective, taken monthly	-0.35	-0.53 — -0.18
