# Supplementary material for: Preference for Service Delivery for Long-Acting Pre-exposure Prophylaxis for HIV Infection Among Pregnant and Breastfeeding Women in South Africa and Botswana
Source: AIDS Behav. 2025 May 21;29(9):2963–75. doi: 10.1007/s10461-025-04751-6 (PMC12432069; doi:10.1007/s10461-025-04751-6)

Supporting information

Supplementary Figure 2. Results of the main discrete choice experiment by age

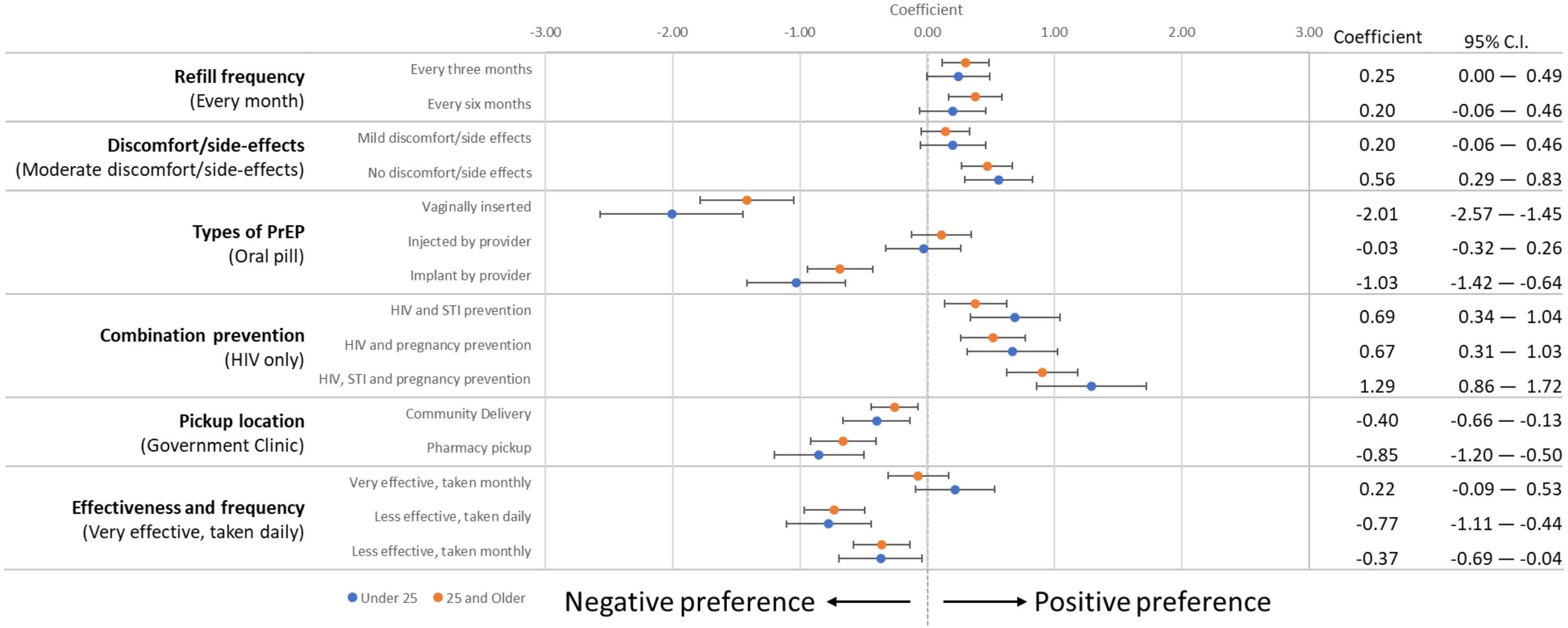

Supplement: Supplementary file 4 — Supplementary Material 4 [file 10461_2025_4751_MOESM4_ESM.pdf]
