# Supplementary material for: Preference for Service Delivery for Long-Acting Pre-exposure Prophylaxis for HIV Infection Among Pregnant and Breastfeeding Women in South Africa and Botswana
Source: AIDS Behav. 2025 May 21;29(9):2963–75. doi: 10.1007/s10461-025-04751-6 (PMC12432069; doi:10.1007/s10461-025-04751-6)
Supplement: Supplementary file 5 — Supplementary Material 5 [file 10461_2025_4751_MOESM5_ESM.pdf]

Supplementary Information

Supplemental Table 3. Standard deviation estimates: Coefficients, p-values and 95% confidence intervals derived from the PrEP-CHOICE discrete choice experiment (by site)

1. CAPE TOWN

Attribute	Level	Coefficient	Std error	P-value	95% confidence interval	
Refill frequency (Every month)	Every three months	0.19	0.54	0.728	-0.87	1.25
	Every six months*	0.73	0.37	**0.049**	0.00	1.47
Discomfort/side-effects (Moderate)	Mild discomfort/side effects*	0.69	0.30	**0.023**	0.10	1.29
	No discomfort/side effects	0.00	0.61	0.995	-1.20	1.21
Types of PrEP (Oral pill)	Vaginally inserted**	2.69	0.54	**<0.001**	1.63	3.76
	Injected by provider**	1.50	0.37	**<0.001**	0.77	2.23
	Implant by provider**	1.59	0.37	**<0.001**	0.87	2.31
Combination prevention (HIV only)	HIV and STI prevention	0.79	0.38	0.037	0.05	1.53
	HIV and pregnancy prevention**	-0.68	0.38	0.076	-1.44	0.07
	HIV, STI and pregnancy prevention**	0.54	0.42	0.203	-0.29	1.37
Pickup location (Government Clinic)	Community Delivery	0.23	0.40	0.556	-0.54	1.01
	Pharmacy pickup**	1.70	0.36	**<0.001**	0.99	2.42
Effectiveness and frequency	Very effective, taken monthly**	1.37	0.41	**0.001**	0.58	2.16
	Less effective, taken daily	0.07	0.36	0.848	-0.64	0.78

(Very effective, taken daily)	Less effective, taken monthly*	-0.11	0.42	0.795	-0.94	0.72

Bold p<0.5

2. EAST LONDON

Attribute	Level	Coefficient	Std error	P-value	95% confidence interval	
Refill frequency (Every month)	Every three months	-0.01	0.23	0.95	-0.45	0.43
	Every six months**	0.69	0.19	**<0.001**	0.32	1.06
Discomfort/side-effects (Moderate)	Mild discomfort/side effects	-0.23	0.35	0.52	-0.92	0.46
	No discomfort/side effects	-0.02	0.37	0.96	-0.74	0.70
Types of PrEP (Oral pill)	Vaginally inserted**	0.88	0.23	**<0.001**	0.43	1.34
	Injected by provider**	0.96	0.23	**<0.001**	0.50	1.41
	Implant by provider**	1.04	0.23	**<0.001**	0.59	1.49
Combination prevention (HIV only)	HIV and STI prevention*	-0.24	0.42	0.57	-1.07	0.59
	HIV and pregnancy prevention**	-0.11	0.34	0.75	-0.77	0.56
	HIV, STI and pregnancy prevention**	0.42	0.36	0.25	-0.29	1.13
Pickup location (Government Clinic)	Community Delivery	-0.14	0.39	0.72	-0.91	0.63
	Pharmacy pickup**	0.73	0.21	**<0.001**	0.32	1.13
Effectiveness and frequency (Very effective, taken daily)	Very effective, taken monthly	-0.44	0.37	0.23	-1.17	0.28
	Less effective, taken daily*	0.61	0.27	**0.03**	0.08	1.15

	Less effective, taken monthly**	0.78	0.25	**<0.001**	0.28	1.28

3. GABORONE

Attribute	Level	Coefficient	Std error	P-value	95% confidence interval	
Refill frequency (Every month)	Every three months	-0.27	0.39	0.493	-0.01	0.45
	Every six months	0.49	0.40	0.218	-0.06	0.45
Discomfort/side-effects (Moderate)	Mild discomfort/side effects**	-0.14	0.36	0.705	0.09	0.57
	No discomfort/side effects**	0.20	0.45	0.661	0.42	0.94
Types of PrEP (Oral pill)	Vaginally inserted**	2.28	0.55	**<0.001**	-1.47	-0.76
	Injected by provider	-1.02	0.41	0.013	-0.07	0.55
	Implant by provider**	-1.36	0.44	**0.002**	-0.94	-0.27
Combination prevention (HIV only)	HIV and STI prevention*	0.67	0.39	0.089	0.11	0.70
	HIV and pregnancy prevention**	0.62	0.48	0.194	-0.03	0.58
	HIV, STI and pregnancy prevention**	-1.55	0.39	**<0.001**	0.23	0.83
Pickup location (Government Clinic)	Community Delivery	0.44	0.29	0.127	-0.75	-0.25
	Pharmacy pickup**	1.75	0.43	**<0.001**	-0.74	-0.16
Effectiveness and frequency (Very effective, taken daily)	Very effective, taken monthly	0.65	0.40	0.107	-0.31	0.27
	Less effective, taken daily**	1.59	0.41	**<0.001**	-0.92	-0.31

	Less effective, taken monthly**	-1.15	0.39	**0.003**	-0.51	0.09
