# Supplementary material for: Preference for Service Delivery for Long-Acting Pre-exposure Prophylaxis for HIV Infection Among Pregnant and Breastfeeding Women in South Africa and Botswana
Source: AIDS Behav. 2025 May 21;29(9):2963–75. doi: 10.1007/s10461-025-04751-6 (PMC12432069; doi:10.1007/s10461-025-04751-6)
Supplement: Supplementary file 6 — Supplementary Material 6 [file 10461_2025_4751_MOESM6_ESM.pdf]

Supplementary Information

Supplemental Table 4. Standard deviation estimates: Coefficients, p-values and 95% confidence intervals derived from the PrEP-CHOICE discrete choice experiment (by perinatal period)

1. PREGNANT

Attribute	Level	Coefficient	Std error	P-value	95% confidence interval	
Refill frequency (Every month)	Every three months	0.14	0.38	0.709	-0.87	1.25
	Every six months	0.44	0.24	0.071	0.00	1.47
Discomfort/side-effects (Moderate)	Mild discomfort/side effects	-0.29	0.33	0.38	0.10	1.29
	No discomfort/side effects	0.20	0.43	0.644	-1.20	1.21
Types of PrEP (Oral pill)	Vaginally inserted**	1.70	0.25	**<0.001**	1.63	3.76
	Injected by provider**	1.14	0.23	**<0.001**	0.77	2.23
	Implant by provider**	0.85	0.29	**0.003**	0.87	2.31
Combination prevention (HIV only)	HIV and STI prevention	0.04	0.49	0.935	0.05	1.53
	HIV and pregnancy prevention*	0.67	0.26	**0.01**	-1.44	0.07
	HIV, STI and pregnancy prevention*	0.58	0.24	**0.015**	-0.29	1.37
Pickup location (Government Clinic)	Community Delivery	-0.12	0.44	0.79	-0.54	1.01
	Pharmacy pickup**	1.15	0.20	**<0.001**	0.99	2.42
Effectiveness and frequency (Very effective, taken daily)	Very effective, taken monthly	-0.52	0.31	0.095	0.58	2.16
	Less effective, taken daily*	0.67	0.30	**0.024**	-0.64	0.78

	Less effective, taken monthly**	0.81	0.24	**0.001**	-0.94	0.72

Bold p<0.5

2. POSTPARTUM/BREASTFEEDING

Attribute	Level	Coefficient	Std error	P-value	95% confidence interval	
Refill frequency (Every month)	Every three months	0.00	0.23	0.994	-0.46	0.45
	Every six months**	0.72	0.20	**<0.001**	0.32	1.11
Discomfort/side-effects (Moderate)	Mild discomfort/side effects	-0.03	0.45	0.939	-0.92	0.85
	No discomfort/side effects	0.21	0.29	0.46	-0.35	0.77
Types of PrEP (Oral pill)	Vaginally inserted**	1.61	0.25	**<0.001**	1.12	2.09
	Injected by provider**	0.82	0.22	**<0.001**	0.39	1.26
	Implant by provider**	1.15	0.20	**<0.001**	0.75	1.55
Combination prevention (HIV only)	HIV and STI prevention	0.03	0.35	0.938	-0.67	0.72
	HIV and pregnancy prevention	0.00	0.23	0.984	-0.45	0.44
	HIV, STI and pregnancy prevention**	1.10	0.23	**<0.001**	0.66	1.54
Pickup location (Government Clinic)	Community Delivery*	0.53	0.21	**0.01**	0.13	0.94
	Pharmacy pickup**	0.95	0.19	**<0.001**	0.58	1.31
Effectiveness and frequency	Very effective, taken monthly*	0.63	0.27	**0.019**	0.11	1.15
	Less effective, taken daily	-0.37	0.35	0.288	-1.04	0.31

(Very effective, taken daily)	Less effective, taken monthly	0.36	0.32	0.274	-0.28	0.99
