# Supplementary material for: Preference for Service Delivery for Long-Acting Pre-exposure Prophylaxis for HIV Infection Among Pregnant and Breastfeeding Women in South Africa and Botswana
Source: AIDS Behav. 2025 May 21;29(9):2963–75. doi: 10.1007/s10461-025-04751-6 (PMC12432069; doi:10.1007/s10461-025-04751-6)
Supplement: Supplementary file 7 — Supplementary Material 7 [file 10461_2025_4751_MOESM7_ESM.pdf]

<u>**Supplementary Information**</u>

Supplemental Table 5. Standard deviation estimates: Coefficients, p-values and 95% confidence intervals derived from the PrEP-CHOICE discrete choice experiment (by age)

1. < 25 YEARS

Attribute	Level	Coefficient	Std error	P-value	95% confidence interval	
Refill frequency (Every month)	Every three months	0.01	0.26	0.959	-0.50	0.52
	Every six months	0.30	0.52	0.568	-0.73	1.32
Discomfort/side-effects (Moderate)	Mild discomfort/side effects	0.48	0.28	0.081	-0.06	1.02
	No discomfort/side effects	0.10	0.33	0.757	-0.54	0.75
Types of PrEP (Oral pill)	Vaginally inserted**	1.48	0.27	**<0.001**	0.96	2.00
	Injected by provider**	0.95	0.27	**<0.001**	0.42	1.47
	Implant by provider**	1.15	0.24	**<0.001**	0.68	1.62
Combination prevention (HIV only)	HIV and STI prevention**	0.81	0.28	**0.003**	0.27	1.35
	HIV and pregnancy prevention	0.14	0.32	0.667	-0.50	0.78
	HIV, STI and pregnancy prevention**	0.83	0.26	**0.001**	0.33	1.33
Pickup location (Government Clinic)	Community Delivery**	0.60	0.22	**0.006**	0.18	1.03
	Pharmacy pickup**	0.95	0.21	**<0.001**	0.54	1.37
Effectiveness and frequency (Very effective, taken daily)	Very effective, taken monthly*	0.71	0.29	**0.016**	0.13	1.29
	Less effective, taken daily*	-0.61	0.28	**0.029**	-1.17	-0.06

	Less effective, taken monthly**	0.71	0.32	**0.026**	0.08	1.33

Bold p<0.5

2. ≥ 25 YEARS

Attribute	Level	Coefficient	Std error	P-value	95% confidence interval	
Refill frequency (Every month)	Every three months	-0.02	0.35	0.95	-0.71	0.67
	Every six months**	0.56	0.18	**0.002**	0.21	0.90
Discomfort/side-effects (Moderate)	Mild discomfort/side effects	-0.14	0.29	0.644	-0.71	0.44
	No discomfort/side effects	0.12	0.40	0.766	-0.66	0.89
Types of PrEP (Oral pill)	Vaginally inserted**	1.77	0.23	**<0.001**	1.32	2.22
	Injected by provider**	0.95	0.19	**<0.001**	0.57	1.32
	Implant by provider**	0.93	0.19	**<0.001**	0.55	1.31
Combination prevention (HIV only)	HIV and STI prevention	-0.09	0.26	0.747	-0.60	0.43
	HIV and pregnancy prevention	0.27	0.36	0.462	-0.44	0.97
	HIV, STI and pregnancy prevention**	0.71	0.21	**0.001**	0.30	1.12
Pickup location (Government Clinic)	Community Delivery	-0.17	0.25	0.486	-0.65	0.31
	Pharmacy pickup**	1.10	0.17	**<0.001**	0.78	1.43
Effectiveness and frequency	Very effective, taken monthly**	0.63	0.21	**0.003**	0.21	1.05
	Less effective, taken daily	-0.49	0.30	0.101	-1.08	0.10

(Very effective, taken daily)	Less effective, taken monthly	-0.36	0.27	0.179	-0.88	0.16
